# Efficacy of Internet-Based Guided Treatment for Genito-Pelvic Pain/Penetration Disorder: Rationale, Treatment Protocol, and Design of a Randomized Controlled Trial

**DOI:** 10.3389/fpsyt.2017.00260

**Published:** 2018-01-22

**Authors:** Anna-Carlotta Zarski, Matthias Berking, David Daniel Ebert

**Affiliations:** ^1^Department of Clinical Psychology and Psychotherapy, Friedrich-Alexander University Erlangen-Nürnberg, Erlangen, Germany; ^2^Institute of Psychology, Leuphana University Lüneburg, Lüneburg, Germany

**Keywords:** genito-pelvic pain/penetration disorder, vaginismus, dyspareunia, sexual dysfunction, Internet intervention, randomized controlled trial

## Abstract

**Introduction:**

Genito-pelvic pain/penetration disorder (GPPPD) not only adversely affects women’s sexuality and sexual satisfaction but is also associated with a wide range of psychosocial consequences such as reduced quality of life and well-being, mental health comorbidities, and relationship distress. Evidence for effective treatment options is scarce.

**Aim:**

This article describes the rationale, treatment protocol, and study design for a randomized controlled trial examining the efficacy of an Internet-based guided intervention for GPPPD.

**Method:**

Two hundred women who meet the criteria for GPPPD and have not been able to experience sexual intercourse for at least the last 6 months will be recruited and randomly assigned either to the intervention group (IG) or a 6-month waitlist control group. Assessments take place at baseline (T1), peritreatment after completion of Session 5 in IG (T2), after completion of Session 8 or 12 weeks after randomization (T3), and after 6 months (T4). Data will be analyzed on an intention-to-treat and a completer basis.

**Main outcome measures:**

The primary outcome will be sexual intercourse involving the insertion of the partner’s penis at posttreatment. Secondary outcomes include, e.g., improved non-intercourse penetration, sexual functioning, dyadic stress coping, reduced fear of sexuality and negative penetration-related cognitions. Fear of sexuality, penetration-related cognitions, and exercise intensity will be assessed as mediators of intercourse in the IG. Sexual dysfunctions of partners will be measured at baseline (T1) and investigated as a potential moderator of the primary treatment outcome.

**Discussion:**

Given the burden associated with GPPPD and the need for specialized treatment, there is a surprising lack of evidence-based treatment options. This study aims to assess whether Internet-based interventions could contribute to closing this treatment gap.

**Clinical Trial Registration:**

German Register of Clinical Studies (DRKS): DRKS00010228.

## Background

Genito-pelvic pain/penetration disorder (GPPPD) is a new diagnosis included in the 5th edition of the Diagnostic and Statistical Manual of Mental Disorders (DSM-5), which merged the revised definitions of both female sexual dysfunctions dyspareunia and vaginismus (1). At least one of the following persistent or recurrent criteria characterizes GPPPD: (1) difficulties with vaginal penetration during intercourse, (2) genito-pelvic pain during vaginal intercourse or penetration attempts, (3) fear or anxiety associated with genito-pelvic pain or vaginal penetration, or (4) tightness of the pelvic floor muscles during attempted vaginal penetration (1). One or more of these symptoms have to be present for at least 6 months and must cause clinically significant distress. GPPPD can be classified as either lifelong or acquired and, depending on the level of distress as mild, moderate or severe (1). The fusion of vaginismus and dyspareunia under a new classification and set of criteria was due to the significant overlap in clinical presentation, and exceeding difficulties to distinguish between the two reliably (2, 3).

The prevalence of GPPPD has not been ascertained due to the novel criteria set. Reported prevalence rates in the general population vary between 3 and 25% for dyspareunia (4–8) and 0.4 and 6.6% for vaginismus (6, 7, 9). Prevalence estimates are heterogeneous due to, e.g., varying diagnostic criteria, assessment methods, study design, and sample characteristics (10–12).

The burden of suffering associated with GPPPD and linked conditions such as vulvodynia and provoked vestibulodynia is high as symptoms have a detrimental impact on physiological and psychological health, and relational well-being. GPPPD has been shown to have a negative effect on the women’s overall quality of life (13), with 60% of women reporting that the disorder compromised their ability to enjoy life (14). Moreover, it has been linked to depression and anxiety disorder (15–17). GPPPD symptoms are often comorbid with a wide range of other sexual dysfunctions and reduced sexual behavior (18–20). Many women with GPPPD also experience problems when using tampons or during gynecological examinations (21, 22). GPPPD has been shown to contribute to declines in self-esteem and feelings of femininity and is associated with a negative body and genital self-image (23–25). It can pose a considerable burden on a couples’ relationship, especially if they would like to have children (26).

Regarding the broad symptom profile of GPPPD, its etiology and maintenance can be best explained by a biopsychosocial framework considering a wide range of interdependent pathophysiological, psychological, social, cultural, and relational factors as well as critical life events (27). According to the fear-avoidance model (28), maladaptive cognitions including worrying about losing control of one’s body, genital incompatibility, and pain catastrophizing are crucial in maintaining and reinforcing genito-pelvic pain and associated GPPPD symptoms, by leading to fear and hypervigilance in sexual situations, or complete avoidance of sexual intimacy (18, 29, 30). Beyond individual aspects, relationship dynamics such as dyadic communication and stress coping, as well as partners’ responses have been shown to have profound influence on GPPPD symptoms (31–33). To an appreciable extent, GPPPD has also been found to concur with male partners’ sexual dysfunctions (34, 35).

Due to the biopsychosocial nature of GPPPD, a multidimensional integrative treatment approach is needed that targets not only difficulties with vaginal penetration, pain, anxiety, and muscle tightness associated with sexual intercourse but also sexual satisfaction and couple dynamics (36–38). Psychological interventions for vaginismus and dyspareunia include strategies such as pain management, systematic desensitization, cognitive restructuring, pelvic-floor exercises, sensate focus, and mindfulness (39–41). Only few interventions, however, have been empirically tested so far. Of these trials, only few studies have applied randomized controlled trial (RCT) design, most of them with only small sample sizes (42–45). Findings of these RCTs indicate that psychological treatments can result in significant improvement of intercourse penetration ability, decrease in pain during intercourse, and higher levels of sexual functioning (42–45). However, none of these studies targeted all symptoms of GPPPD.

Moreover, the availability of such specialized treatment options in health care is extremely limited (46, 47). Thus, the majority of women with GPPPD do not receive appropriate treatment, which is, alongside the burden for the individual and partner, also associated with economic consequences, such as increased direct health costs including office visits, hospitalization, and medication (48). Many women with GPPPD do, however, not seek help in the first place although feeling severely distressed (49). Reasons therefore include (1) sexual dysfunctions not being regarded as recognized disorders, and instead a taboo subject in society associated with fear of stigmatization (50, 51), (2) self-stigmatization including feelings of shame and guilt (25, 50, 52), (3) health care professionals not standardly inquiring about sexual difficulties in routine care visits due to feelings of inadequacy or embarrassment or due to a lack of training, knowledge, or time (53, 54).

Internet-based interventions can be one strategy to address some of the limitations of traditional psychological interventions with regard to limited availability, high threshold, and costs (55). They (1) are readily available and easily accessible independent of time and place, (2) offer anonymity, (3) allow users to work at their own pace and to review materials as often as they want, (4) are more likely to reach affected women earlier compared with traditional mental health services, hence preventing the onset of other mental health problems, (5) reach women who might otherwise not seek out help (56), and (6) are easily scalable regarding implementation and dissemination. In both community and clinical settings, Internet-based interventions have been shown to be an effective mean to treat other sexual dysfunctions (57–60), anxiety (61, 62) and depression (63–65) amongst other mental health disorders (56). However, to the best of our knowledge, there is no published trial evaluating an Internet-based guided intervention for GPPPD in an RCT design.

### Aims

In this study, we will investigate the efficacy of a newly developed Internet-based guided self-help intervention for GPPPD. We hypothesize that participants in the intervention group (IG) are more likely to achieve intercourse penetration, the primary outcome in this study, from pretest to posttest, compared to a waitlist control group (WCG), and that this effect will also be sustained until a 6-month follow-up. In addition, the study aims (1) to analyze changes concerning secondary outcomes compared to the WCG, (2) to assess predictors and moderators of intercourse penetration and treatment adherence in explorative analyses, (3) to analyze fear of sexuality, penetration-related cognitions, and exercise intensity as mediators of intercourse penetration in the IG, (4) to investigate sexual dysfunctions of partners as a potential moderator of treatment outcome, and (5) to examine patients experiences with the intervention and treatment adherence qualitatively in order to further improve Internet-based interventions for GPPPD.

## Methods

### Design

**Figure 1:**
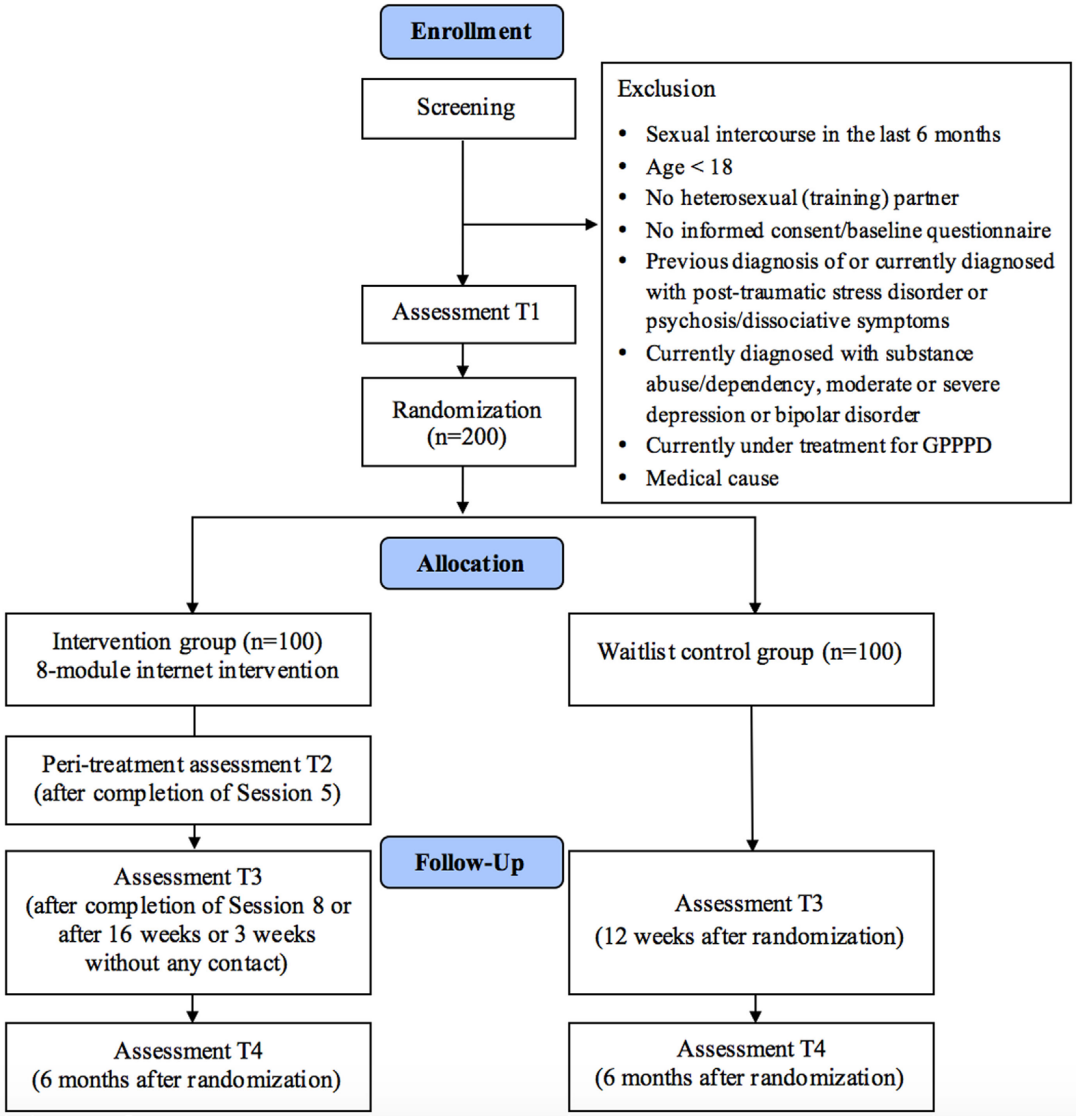
Flow of participants.

An RCT will be conducted to compare the IG (Paivina-Care) with a WCG. Self-reported assessments will take place at baseline (T1), peritreatment (after completion of Session 5, T2 in IG), posttreatment (after completion of Session 8 or 12 weeks after randomization, T3) and at a 6-month follow-up (T4; see Figure 1 for a detailed overview). Self-reported data will be collected using a secure online-based assessment system (AES, 256-bit encrypted). All procedures involved in the study will be consistent with the generally accepted standards of ethical practice. The study was approved by the Friedrich-Alexander University Erlangen-Nürnberg ethics committee (no. 324_15B). The trial is registered in the German clinical trials register under DRKS00010228. All participants gave written informed consent in accordance with the Declaration of Helsinki.

### Participants and Procedure

#### Inclusion and Exclusion Criteria

We will include women who (1) have not been able to have sexual intercourse in at least the last 6 months conformed to DSM-5, as indicated by scores ≤ 1 on Primary Endpoint Questionnaire (PEQ) item 7 (60), (2) are at least 18 years of age, (3) whose penetration difficulties are not due to a medical condition, (4) are in a heterosexual relationship or have a heterosexual (training) partner, (5) who have Internet access, (6) have sufficient German skills in reading and writing, and (7) are willing to give informed consent.

We will exclude individuals reporting to have been diagnosed with (1) psychosis or dissociative symptoms in the past or present, (2) posttraumatic stress disorder or traumatization due to sexual abuse in the past or the present, (3) substance abuse or dependency in the present, or (4) moderate or severe depression or bipolar disorder in the present assessed with the Structured Clinical Interview for Diagnostic and Statistical Manual of Mental Disorders (fourth edition) Axis I Disorders (SCID-I) (66), and those (5) currently being treated for GPPPD.

#### Recruitment

We will recruit patients *via* (1) the study website and Facebook page, (2) articles in women’s online magazines, (3) online forums, (4) advertisements on websites related to the topic of sexual dysfunctions, and (5) Google AdWords. Recruitment is scheduled to take place between April 2016 and December 2017. The trial is open to all women fulfilling the inclusion criteria. The research website www.paivina-care.info provides information on the program and details about the study. If they want to participate in the study, applicants can sign up on this website by leaving their email address.

#### Stepwise Procedure: Assessment of Eligibility and Randomization

Women who sign up as potential participants will receive an online information letter with detailed information on the study procedures. They will be asked to provide an email address that they want to use for the study and a first and last name (which can be pseudonyms if desired). The women will be informed that they can withdraw from the intervention and/or study at any time without any negative consequences. Applicants who intend to participate in the study will be asked to complete online screening questionnaires that assess inclusion and exclusion criteria. If responses to this questionnaire indicate that participants might be affected by one of the mental health disorders listed as exclusion criteria above, participants will be scheduled for the SCID-I *via* telephone. Women who meet all of the inclusion criteria and none of the exclusion criteria and who complete the baseline assessment and return the informed consent form will be considered for the study and randomly allocated to one of the two study conditions. Randomization will take place at an individual level. This process will be completed by an independent researcher not otherwise involved in the study using an automated computer-based random integer generator (randlist). Until this process has been completed, the results will not be known by participants, researchers involved in recruitment or eCoaches. Participants will be informed of the outcome of the randomization, and participants in the IG will receive immediate access to the program. Participants in the WCG will receive access to the program 6 months after study intake.

### Intervention

The intervention consists of eight sessions composed of modules for psychoeducation (Sessions 1–2), cognitive restructuring (Session 2), non-judgmental awareness (Session 2), relaxation exercises (Session 3), attention-focusing for pain-management (Session 3), body exposure and genital self-exploration (Session 4), gradual exposure with fingers (Session 3) and with dilators (Sessions 5–6), sensate focus (Sessions 5–6), preparation exercises for sexual intercourse (Session 7), and relapse prevention (Session 8). A booster session is provided four weeks after the end of the program (Session 9) (see Table 1 for a session overview). The intervention builds on elements of an evaluated cognitive-behavioral intervention for women with vaginismus and the treatment manual for the present intervention (67, 68). Each session can be completed in approximately 45–60 min. We advise participants to do at least one and no more than two sessions per week. Participants can, however, take more time for session completion if needed. Consequently, the intervention lasts about 8–16 weeks (plus a booster session). Lessons consist of general text-based information including a discussion of the rationale for each program component, testimonials as well as interactive elements such as exercises (all sessions), quizzes (Session 1), mp3 audio files (Session 3), video clips (Sessions 1 and 3), and downloadable worksheets (Sessions 1, 3, 4, 5, and 7). The program is adaptive, as the content is tailored to the specific needs of the individual participant by continuously asking participants to choose among various response options. Subsequent content is then tailored to each participant’s response. Participants will have the possibility to rate how useful they find the modules, how easy the modules are understood, how long they take to complete them, and to add additional comments and feedback. Using responsive web-design, participants can follow the program on a computer, tablet, or mobile phone. Participants can also use an integrated read-aloud function to listen to the texts. Difficulties with exercises as well as challenges, obstacles, disappointment, and setbacks that may occur in the course of treatment are addressed in several sessions. Participants are encouraged to keep a daily online training diary to monitor exacerbating and alleviating factors regarding the use of the different techniques in the program. One key feature of the intervention is the focus on transfer tasks (homework assignments), which allow participants to integrate newly acquired strategies and techniques into daily life.

**Table 1:** Content of the GPPPD program.

Intervention content	Session
Psychoeducation I	1
Psychoeducation II, cognitive restructuring, and non-judgmental awareness	2
Muscle and breathing relaxation, pelvic floor relaxation, attention-focusing for pain management, and gradual vaginal exposure with fingers	3
Body exposure and genital self-exploration	4
Gradual vaginal exposure with dilators I and sensate focus I	5
Gradual vaginal exposure with dilators II, sensate focus II, and building sexual desire and arousal	6
Sexual intercourse	7
Plan for the future, relapse prevention	8
Booster session	9

Internet-based interventions have the advantage of directly promoting health behavior change in the daily life of participants, who are supported and encouraged to complete exercises such as progressive muscle relaxation (PMR) and dilator insertion. In each session, participants are introduced to a new or a follow-up strategy which then has to be practiced in everyday life. The use and implementation of the transfer tasks is then reviewed at the beginning of each session and participants are encouraged to continue exercising each task throughout the intervention. The implementation of the transfer tasks is also supported by text messages on the smartphone including motivational messages and practice reminders. These messages are meant to encourage participants to apply what they have learned in their daily lives. If desired, participants will receive one text message every day during an 8-week period.

The secure web-based platform (AES 256-bit encryption) mentioned above will also be used for the intervention and the communication between participants and eCoaches. Participants will access the platform using their email address and chosen password.

#### Psychoeducation (Sessions 1–2)

The participants will be provided with psychoeducational information on GPPPD and the multifactorial causes of its development. Core elements in the first session are (1) overview of the program: intervention components and the expectations placed on the participants are presented; (2) goal setting: participants are asked to define realistic individual treatment goals, which will be shown and can be modified over the course of the program; (3) strengthening motivation: personal treatment motivation is assessed, participants can schedule the exercises and plan how to deal with difficulties they may face during treatment, for example non-adherence to treatment and exercises; (4) self-support: participants learn about effective support, e.g., in terms of self-gratification; (5) couples’ communication: rules for communication are introduced to facilitate the discussion between participants and their partners about the intervention and the partner’s role in the program, especially with regard to exercises, the expression of emotional needs, and sexual preferences.

In the second part of psychoeducation (Session 2), participants will be introduced to the fear-avoidance model and thereby learn more about the causal and maintaining factors of GPPPD and the rationale behind the treatment. Participants are given the opportunity to individualize their own fear-avoidance model by including their cognitions, emotions, physical reactions, and behaviors. Participants also learn about the function of emotions and their influence on GPPPD symptoms.

#### Cognitive Restructuring (Session 2)

In the first step, participants identify negative cognitions and distorted beliefs about sexual intercourse, genital pain, and sexuality in general, and learn how cognitions can influence emotions and behavior. In the second step, they restructure and replace these cognitions with more helpful and encouraging ones. These cognitions are also rehearsed as coping self-statements during the exercises.

#### Non-Judgmental Awareness (Session 2)

By learning about and practicing non-judgmental awareness, participants might be able to interrupt automatic processes of negative cognitions. They are instructed to observe their cognitions and emotions in response to sexual situations without interpreting, judging, reacting to or suppressing them. To get used to the principles of non-judgmental awareness, participants are asked to practice it not only in sexual but also in nonsexual day-to-day situations.

#### Muscle and Breathing Relaxation (Session 3)

Participants will learn PMR in combination with breathing relaxation (69). By learning to flex and relax muscles voluntarily, participants can begin to control their body tension. Guided by an audio file, they start to practice PMR and eventually switch to a shorter version that can also be integrated into day-to-day activities. Based on the principles of PMR, pelvic floor exercises are then introduced in a video tutorial. These exercises are meant to increase proprioception and decrease hypertonicity of the pelvic floor.

#### Attention-Focusing for Pain Management (Session 3)

In Session 3, participants are also introduced to the relationship between vaginal pain and anxiety, and the consequences of pain and fear of pain on sexual arousal and muscle tension. They are asked to explore exacerbating and alleviating factors that influence their genito-pelvic pain. During attention-focusing, patients localize the pain center, describe the pain as images, and use suggestive pain transformation to alter the pain.

#### Body Exposure and Genital Self-Exploration (Session 4)

The internal and external female genitals and the anatomy of pelvic floor are introduced and illustrated with drawing. This treatment component also addresses the physiological processes during arousal and sexual intercourse. Participants are introduced to a body exposure exercise and asked to explore their vagina in front of a mirror. The approach taken here is non-judgmental and mindful of negative cognitions and emotions. Genital self-exploration is complemented by other, shorter body exposure exercises that are meant to enhance participants’ positive self-perception of their bodies and desirability.

#### Sensate Focus (Session 4)

The aim of the sensate focus exercises is to promote physical intimacy and reduce associated stress, pressure, and anxiety by emphasizing the experience of sensual pleasure by the couple over intercourse or orgasm (70, 71). Sensate focus exercises foster sensual body awareness and allow participants to become better acquainted with their own and their partners’ bodies and erogenous zones. These exercises involve progressive steps in which partners touch and give massages to each other (1) first nongenitally, wearing clothes and then only underwear, (2) with genital touching but not aiming for sexual arousal, and (3) genital touching that can lead to sexual arousal. During the exercises, the participants and their partners switch active and passive roles and are asked to communicate their feelings and experiences to learn more about their own and their partner’s sensual and sexual pleasures.

#### Building Sexual Desire and Arousal (Session 6)

One of the goals of the treatment is to increase sexual desire and arousal, as both influence vaginal penetration and pain. Information on self-stimulation and masturbation, e.g., techniques, is provided.

#### Gradual Vaginal Exposure (Sessions 3, 5–7)

Gradual vaginal exposure is combined with PMR and pelvic floor exercises. The exercises involve (1) insertion of one and two fingers by the woman and (2) one and two fingers by the partner, whereby the woman guides the partner’s hand while inserting his finger or fingers, (3) self-insertion of dilators of increasing sizes, with the largest dilator being similar in size to a penis, and (4) insertion of dilators of increasing sizes by the partner guided by the woman. Additionally, participants are encouraged to keep the dilator inside the vagina for a longer period of time (up to an hour) to get used to the feeling and to increase the habituation effect. Participants are advised to choose a larger dilator if they are comfortable with repeated insertions. These gradual exposure exercises are accompanied by detailed step-by-step instructions and also include, for example, suggestions for preparing for the exercises, sitting postures and recommendations that will help women deal with possible difficulties.

#### Sexual Intercourse (Session 7)

Step-by-step insertion exercises with the penis include (1) touching the vagina with the erect penis without insertion, (2) inserting the erect penis without moving, and (3) moving the erect penis inside the vagina.

#### Plan for the Future (Session 8)

In the last session, participants will be asked to reassess their training goals and to identify their personal warning signs for GPPPD setbacks. Additionally, they will be given the opportunity to write a letter to themselves describing their life after 4 more weeks of exercises, which they can refer to in the future if they wish.

#### Booster Session

Four weeks after completing the intervention, participants will be given the option of completing a booster session and evaluating their training progress. They will revisit the letter they wrote to themselves in the last session, reassess their goals, and make plans concerning insertion exercises or sexual activities.

### Support

Participants are supported by a personal eCoach. The focus of the coaching is to reinforce treatment efforts, answer questions, and to motivate participants. The eCoaches are female psychologists or trained and supervised psychology students, and will follow guidelines outlining the feedback process as defined in the standardized manual for the intervention. The eCoaches will send reminders in cases in which the participants do not complete one session within 7 days. After each session, participants will receive personalized written feedback on the exercises they have completed from the eCoach within 48 hours. The eCoach also closely monitors and reacts to potential signs of deterioration or other potentially adverse events. Guidelines for feedback follow principles that have been employed in a large number of previous studies from our research group (56, 72).

### Primary and Secondary Outcomes

Primary outcome will be sexual intercourse at posttreatment. In secondary analyses, the effects of the intervention, e.g., on nonintercourse penetration behavior, fear of sexuality, sexual functioning, and cognitions regarding vaginal penetration, will be examined.

### Outcome Measures

#### Intercourse Penetration

The seven-item PEQ will be used as a primary outcome measure. Successful intercourse penetration (SIP) will be measured by one item assessing the degree to which the insertion of the partner’s penis was possible. The items will be answered on a 4-point Likert scale (0 = not attempted; 1 = attempted, but unsuccessful; 2 = attempted and sometimes successful; 3 = attempted and always successful). Previous cases of women reaching SIP prior to treatment completion have shown that they are susceptible to study dropout (67). For that reason, participants who do not fill out the PEQ online, (1) are scheduled for a telephone assessment or (2) can report to their eCoach whether intercourse penetration was successful.

#### Nonintercourse Penetration

Successful nonintercourse penetration will be measured by the remaining six items of the seven-item PEQ. These items assess the degree to which insertion of one or two fingers or another object such as a tampon or a dilator by the woman or her partner was possible. Participants will be asked to answer questions referring to the past two weeks. Cronbach alpha was found to be good (α = 0.81) for this scale (67).

#### Fear of Sexuality

The frequency of a woman’s fear experienced in different sexual situations will be measured using the Fear of Sexuality Questionnaire (FSQ) (73). The FSQ consists of the five-item subscale fear of noncoital sexual activity and the three-item subscale fear of coitus. The items are answered on a 5-point Likert scale ranging from 1 = never to 5 = always. The internal consistency of this measure has been found to be good with a Cronbach alpha of α = 0.82–0.84 for fear of noncoital sexual activity and α = 0.82–0.86 for fear of coitus in samples of women with vaginismus (67, 73).

#### Cognitions Regarding Vaginal Penetration

Vaginal penetration cognitions will be assessed with the Vaginal Penetration Cognition Questionnaire (VPCQ) (74), which is comprised of the five subscales: control, catastrophic and pain, self-image, positive, and genital incompatibility cognition. This self-report instrument uses a 7-point Likert scale anchored by 0 = not at all applicable and 6 = very strongly applicable. Reliability of these five VPCQ subscales ranges from α = 0.70 to α = 0.83 in a sample of women with vaginismus and dyspareunia.

#### Sexual Functioning

To measure different domains of female sexual functioning, the Female Sexual Function Index will be used (75–77). This self-report instrument consists of the following 6 subscales: sexual desire, sexual arousal, lubrication, orgasm, pain and sexual satisfaction. The questionnaire uses a 6-point Likert scale anchored by 0 = no sexual activity and 5. The measure allows for the calculation of specific indexes for each dimension and of an overall sexual function index. The internal consistency ranges from α = 0.82 to α = 0.93 in women with female sexual arousal disorder (76) and from α = 0.76 to α = 0.93 in a sample of women with vaginal penetration difficulties (67).

#### Satisfaction with the Intervention

Patient satisfaction with the intervention will be measured by using an adaptation of the Client Satisfaction Questionnaire (CSQ-8, German: ZUF-8) optimized for the assessment of client satisfaction with Internet interventions (CSWIQ-8) (78, 79). The CSQ-8 is a validated eight-item instrument with high internal consistency (α = 0.93) (79). The adapted version, validated for the assessment of client satisfaction in Internet-based interventions, has repeatedly proven to have high internal consistency (α = 0.92–0.94) (56, 80, 81) and is associated with treatment adherence and outcome (80).

#### Other Measures

Other measurements include the Structured Clinical Interview for DSM-IV Axis I Disorders (SCID-I), socio-demographic variables (e.g., age, gender, duration of symptoms, etc.), the Well-Being Index (WHO-5) (82–84), the Self-Esteem Scale (85, 86), and the Health Action Process Approach Questionnaire (87) adapted to treatment adherence. Experiences of sexual abuse will be measured with the sexual abuse subscale of the Childhood Trauma Questionnaire (88, 89) and an item adapted to experiences following the age of 18. To assess relationship quality, we will use the Dyadic Coping Inventory (90, 91) and the Partnership Questionnaire Short Form (92). The State Trait Anxiety Inventory (93, 94) and the Generalized Anxiety Disorder 7-item Scale (GAD-7) (95, 96) will be used to assess general anxiety sensibility. Potential negative effects of the Internet-based intervention will be assessed using the Inventory for the Assessment of Negative Effects in Psychotherapy (97). We will also measure potential dropout reasons and the utilization of additional help with self-developed items. Participants will need approximately 40 min to complete all questionnaires at baseline and 30 min at follow-ups. For an overview of all outcome measures, see Table 2.

**Table 2:** Measures.

	T0	T1	T2	T3	T4
Primary Endpoint Questionnaire	✓	✓	✓	✓	✓
Fear of Sexuality Questionnaire	–	✓	✓	✓	✓
Vaginal Penetration Cognition Questionnaire	–	✓	✓	✓	✓
Female Sexual Function Scale	–	✓	–	✓	✓
**Other measurements**					
Socio-Demographic Questionnaire	✓	–	–	–	–
Childhood Trauma Questionnaire	–	✓	–	–	–
Dyadic Coping Inventory	–	✓	–	✓	✓
Partnership Questionnaire Short Form	–	✓	–	✓	✓
State Trait Anxiety Inventory	–	✓	–	✓	✓
Generalized Anxiety Disorder 7-Item Scale	–	✓	–	✓	✓
Well-Being Index	–	✓	–	✓	✓
Self-Esteem Scale	–	✓	–	–	–
Health Action Process Approach Questionnaire	–	✓	✓	–	–
Client Satisfaction Questionnaire	–	–	–	✓	–
Inventory for the Assessment of Negative Effects in Psychotherapy	–	–	–	✓	✓
Regular practicing of the exercises	–	–	–	✓	✓
Potential dropout reasons	–	–	–	✓	✓
Utilization of additional help	–	–	–	✓	✓
**Questionnaires for the partner**					
Socio-Demographic Questionnaire	–	✓	–	–	–
International Index of Erectile Function for Men	–	✓	–	–	–

*T0 = screening, T1 = baseline, T2 = after Session 5 assessed in IG, T3 = posttreatment, T4 = 6-month follow-up*.

#### Sexual Dysfunction Disorders of the Partners

Sexual dysfunction disorders of the partners will be examined using the German version of the International Index of Erectile Function for Men (98, 99). Including 15 items, the questionnaire consists of five subscales: erectile function, orgasmic function, sexual desire, intercourse satisfaction, and overall satisfaction. The first 10 items are rated on a 6-point scale (0–5) with 0 = no intercourse (attempt) and the remaining 5 items are presented with 5-point scales (1–5). High internal consistency has been reported for all subscales with Cronbach alpha between α = 0.71 and α = 0.96 (98, 99).

#### Adherence Measures

Adherence will be assessed by session completion (0–8) tracked by the system of the intervention platform. Participants will also be asked to report how often they completed the exercises in a given week with one item measured on a 4-point Likert scale ranging from 0 = less than once a week to 3 = every day.

### Qualitative Interviews

The aim of the qualitative interview will be the qualitative assessment of factors regarding the intervention efficacy and adherence in order to further improve Internet-based interventions for GPPPD. Telephone interviews will be conducted with all willing intervention completers as well as participants who ceased to complete the intervention. The interview assesses different aspects with regard to Internet-based GPPPD treatment including (1) participants’ expectations and hope of improvement, (2) perceived usefulness of the treatment, (3) reasons for treatment success and failure, (4) reasons for treatment adherence and non-adherence, (5) usability and layout, (6) quality and content of the intervention and the guidance, and (7) overall strengths and limitations of the program. The evaluation relies on qualitative content analyses by Mayring (100) and Kuckartz et al. (101).

### Sample Size Calculation

We aim to include 200 participants to statistically detect a minimal clinical relevant effect size of *w* = 0.20, with a power (1 − β) of 80% and an alpha of 0.05 (two-tailed test) for an intention-to-treat analysis using G*Power. The design of the study is based on the expected superiority of the IG compared to the WCG on the primary outcome variable (i.e., sexual intercourse) at posttreatment (T3).

### Statistical Analyses

#### Clinical Effectiveness

Analyses will be conducted and reported in accordance with the statement by Consolidated Standards of Reporting Trials (102). Data will be analyzed on an intention-to-treat basis. Additionally, study completer analyses including only participants who filled out the questionnaires and intervention completer analyses including only participants who have completed the 8 treatment sessions will be conducted. Missing data will be handled using multiple imputations (MIs) calculated by a Markov Chain Monte Carlo multivariate imputation algorithm of 100 estimations per missing value. MI is especially robust with respect to missing data (103). We will use chi-square test (χ^2^) to examine differences in the primary outcome. Differences in secondary outcome measures will be analyzed between and within the two study groups based on regression. For all analyses, Cohen’s *d* (104), including 95% confidence intervals, will be calculated for the differences between baseline and follow-up scores calculated by using the mean difference in change between the IG and WCG divided by the pooled standard deviation of the change score. For all statistical analyses, the significance level will be set at p < 0.05, 2-sided. All analyses will be performed with IBM SPSS v. 24.

#### Predictor, Moderator, and Mediator Analyses

Predictors and moderators of changes in SIP will be analyzed on an exploratory basis. They will include, e.g., lifelong and acquired GPPPD, experiences of sexual abuse, and symptom severity. Fear of sexuality, penetration-related cognitions and exercise intensity will be examined as mediators of changes in SIP *via* structural equation modeling. Separate moderator analyses will be conducted using the PROCESS macro for SPSS. The analyses will include the main effect of the baseline variable, the main effect of the treatment condition (IG and WCG), and the interaction of the baseline variable and the treatment condition. To assess relevant subgroup effects, follow-up simple slope analyses for possible significant interaction effects will be conducted. Therefore, the slope and the significance of the intervention main effect will be evaluated for conditional values (one SD below and one SD above the mean) of the moderator.

## Discussion

Genito-pelvic pain/penetration disorder is a major strain on afflicted women and their partners and adversely affects sexual satisfaction, sexual functioning, physiological, and psychological well-being. One study showed that treatment involving exposure and face-to-face guidance by a therapist can be an effective means to treat lifelong vaginismus (105). This kind of treatment is, however, very resource-intensive, and access is also limited. Thus, Internet-based guided self-help interventions drawing on the principles of multimodal cognitive-behavioral therapy could be an effective first step in stepped treatments of GPPPD because they are likely to lower the threshold of treatment utilization due to higher levels of anonymity. As a result, women reluctant or unable to participate in face-to-face interventions would be able to seek treatment. Internet-based interventions would also be an option for women at an early stage or with less severe symptoms of the disorder, thus perhaps preventing a chronic course of GPPPD.

Throughout the treatment, participants are supported by an eCoach as guidance, such as that provided in this study, has been shown to increase adherence rates when it comes to Internet-based interventions (106). To increase treatment adherence, eCoaches will send participants feedback on completed treatment sessions, and they will be available for consultation, supporting participants as they, e.g., perform insertion exercises. To prevent a drop in adherence rates due to anticipatory anxiety during the course of treatment, exposure elements such as the insertion of one and two fingers are integrated into the intervention at an early stage. In a pilot trial evaluating an Internet-based intervention for women with vaginal penetration difficulties, the insertion exercises were introduced at the last stage of the intervention, and sessions including insertion exercises were associated with substantial non-adherence (67). The present study will evaluate the acceptability and efficacy of the newly developed intervention, providing valuable information on CBT treatment of GPPPD.

### Anticipated Results

We anticipate an increase in the ability to have sexual intercourse as well as positive cognitions toward vaginal penetration and intercourse in the IG compared to the WCG. A decrease is expected in genito-pelvic pain, fear of pain and penetration, and negative cognitions associated with intercourse and sexuality in the IG not in the WCG. With regard to potential pitfalls, recruiting the sample of women with GPPPD is a major challenge because the majority of them might not have contacted a health practitioner previously for their sexual dysfunction. Moreover, nonadherence to treatment is a common problem not only in Internet-based interventions but in psychotherapy in general. Especially in women with pain-penetration disorder, vaginal insertion is often associated with fear and avoidance behavior, increasing the likelihood for nonadherence to treatment despite the guidance included (67). As a troubleshooting strategy, we will include extensive adherence reminders *via* email and telephone in addition to guidance by an eCoach, which has been shown to be linked to higher treatment adherence in Internet-based interventions (106). The eCoach also closely monitors potential adverse effects in patients. If, contrary to expectations, participants experience a deterioration of symptoms or other adverse effects, the eCoach offers additional support and provides information on how to gain access to other treatment options. Additionally, participants can access information on further treatment options, e.g., face-to-face therapy, in module 8. Moreover, we assess potential negative effects with an evaluated questionnaire in a standardized procedure (97).

### Strengths

This study will contribute significantly to the literature and empirically tested treatments for GPPPD because, to the best of our knowledge, it is the first to investigate the efficacy of a guided Internet-based intervention for the treatment of GPPPD. A major strength of this study is its robust RCT design, commonly regarded as the gold standard for clinical trials. We will also include a high number of participants in this study to ensure sufficient power. In addition, missing data will be handled with MIs, and statistical analyses will be conducted with state-of-the-art methods. Another major advantage of this study is that it also includes a questionnaire on male sexual dysfunction, which the partners of participants will complete. The inclusion of this questionnaire will allow for an analysis of the influence of partners’ sexual disorders on participants’ treatment progress. Moreover, little is known about other moderators and mediators of the treatment outcome for GPPPD. In this study, potential moderators and mediators will be analyzed to understand for which women Internet-based interventions are likely to be the most effective and under what circumstances the intervention can be optimized for participants based on personal characteristics.

### Limitations

This study has the following limitations. First, the present study only includes women who have not been able to experience sexual intercourse for at least 6 months. Thus, our findings may not apply to women who are still able to have sexual intercourse but experience pain. Second, we will not include any objective measurement of GPPPD, e.g., gynecological examinations. Due to feasibility limitations, only self-reported measurements will be considered here. However, initial gynecological examinations are strongly recommended to make sure that the symptoms described by participants are not due to a medical condition. Future studies should focus on the development and evaluation of a diagnostic instrument for GPPPD which assesses all four dimensions in detail as well as distress associated with the condition.

## Conclusion

Overall, to complement research on GPPPD and to advance evidence-based treatment options, RCTs evaluating treatments for GPPPD are needed. Internet-based interventions might be a promising strategy to overcome some of the limitations of current treatment options including limited availability. This study will contribute to empirical research on GPPPD interventions and provide information about their acceptability and efficacy. It is, however, not yet clear which component of the multimodal cognitive-behavioral therapy is most important in treating GPPPD. If successful, this intervention could be made available to a large number of women because of the low threshold, lower costs and accessibility irrespective of place and time.

## Current Status

Recruitment began on April 1, 2016, and will continue approximately through December 31, 2017.

## Ethics Statement

This study has been approved by the ethics committee of the Friedrich-Alexander University Erlangen-Nürnberg (no. 324_15B).

## Author Contributions

A-CZ and DE developed the Paivina-Care Internet-based treatment and designed the study, MB contributed to the study design. A-CZ drafted the manuscript supervised by DE, and MB contributed to the further writing of the manuscript. All authors read and approved the final manuscript and agreed to be accountable for all aspects of the work in ensuring that questions related to the accuracy or integrity of any part of the work are appropriately investigated and resolved.

## Conflict of Interest Statement

DE and MB are stakeholders of the “Institute for Online Health Trainings” that aims to transfer scientific knowledge into routine healthcare. All other authors declare that the research was conducted in the absence of any commercial or financial relationships that could be construed as a potential conflict of interest.
